# Comparison of novel and standard diagnostic tools for the detection of *Schistosoma mekongi* infection in Lao People’s Democratic Republic and Cambodia

**DOI:** 10.1186/s40249-017-0335-x

**Published:** 2017-08-10

**Authors:** Youthanavanh Vonghachack, Somphou Sayasone, Virak Khieu, Robert Bergquist, Govert J. van Dam, Pytsje T. Hoekstra, Paul L. A. M. Corstjens, Beatrice Nickel, Hanspeter Marti, Jürg Utzinger, Sinuon Muth, Peter Odermatt

**Affiliations:** 1 Faculty of Basic Sciences, University of Health Sciences, Ministry of Health, Vientiane, Lao People’s Democratic Republic; 2 Swiss Tropical and Public Health Institute, P.O. Box, CH-4002 Basel, Switzerland; 3 University of Basel, P.O. Box, CH-4003 Basel, Switzerland; 4 National Institute of Public Health, Ministry of Health, Vientiane, Lao People’s Democratic Republic; 5 National Centre for Parasitology, Entomology and Malaria Control, Ministry of Health, Phnom Penh, Cambodia; 6 Ingerod, Brastad, Sweden; 7 Department of Parasitology, Leiden University Medical Center, Leiden, The Netherlands; 8 Department of Molecular Cell Biology, Leiden University Medical Center, Leiden, The Netherlands

**Keywords:** Cambodia, Food-borne trematodes, Kato-Katz, Lao People’s Democratic Republic, Point-of-care circulating cathodic antigen, *Schistosoma mekongi*, Serology, Soil-transmitted helminths, Up-converting phosphor-lateral-flow circulating anodic antigen

## Abstract

**Background:**

Given the restricted distribution of *Schistosoma mekongi* in one province in Lao People’s Democratic Republic (Lao PDR) and two provinces in Cambodia, together with progress of the national control programmes aimed at reducing morbidity and infection prevalence, the elimination of schistosomiasis mekongi seems feasible. However, sensitive diagnostic tools will be required to determine whether elimination has been achieved. We compared several standard and novel diagnostic tools in *S. mekongi*-endemic areas.

**Methods:**

The prevalence and infection intensity of *S. mekongi* were evaluated in 377 study participants from four villages in the endemic areas in Lao PDR and Cambodia using Kato-Katz stool examination, antibody detection based on an enzyme-linked immunosorbent assay (ELISA) and schistosome circulating antigen detection by lateral-flow tests. Two highly sensitive test systems for the detection of cathodic and anodic circulating antigens (CCA, CAA) in urine and serum were utilized.

**Results:**

Stool microscopy revealed an overall prevalence of *S. mekongi* of 6.4% (one case in Cambodia and 23 cases in Lao PDR), while that of *Opisthorchis viverrini*, hookworm, *Trichuris trichiura*, *Ascaris lumbricoides* and *Taenia* spp. were 50.4%, 28.1%, 3.5%, 0.3% and 1.9%, respectively. In the urine samples, the tests for CCA and CAA detected *S. mekongi* infections in 21.0% and 38.7% of the study participants, respectively. In the serum samples, the CAA assay revealed a prevalence of 32.4%, while a combination of the CAA assay in serum and in urine revealed a prevalence of 43.2%. There was a difference between the two study locations with a higher prevalence reached in the samples from Lao PDR.

**Conclusions:**

The CCA, CAA and ELISA results showed substantially higher prevalence estimates for *S. mekongi* compared to Kato-Katz thick smears. Active schistosomiasis mekongi in Lao PDR and Cambodia might thus have been considerably underestimated previously. Hence, sustained control efforts are still needed to break transmission of *S. mekongi*. The pivotal role of highly sensitive diagnostic assays in areas targeting elimination cannot be overemphasised.

**Electronic supplementary material:**

The online version of this article (doi:10.1186/s40249-017-0335-x) contains supplementary material, which is available to authorized users.

## Multilingual abstracts

Please see Additional file 1 for translations of the abstract into the five of the six, official working languages of the United Nations.

## Background

**Fig. 1:**
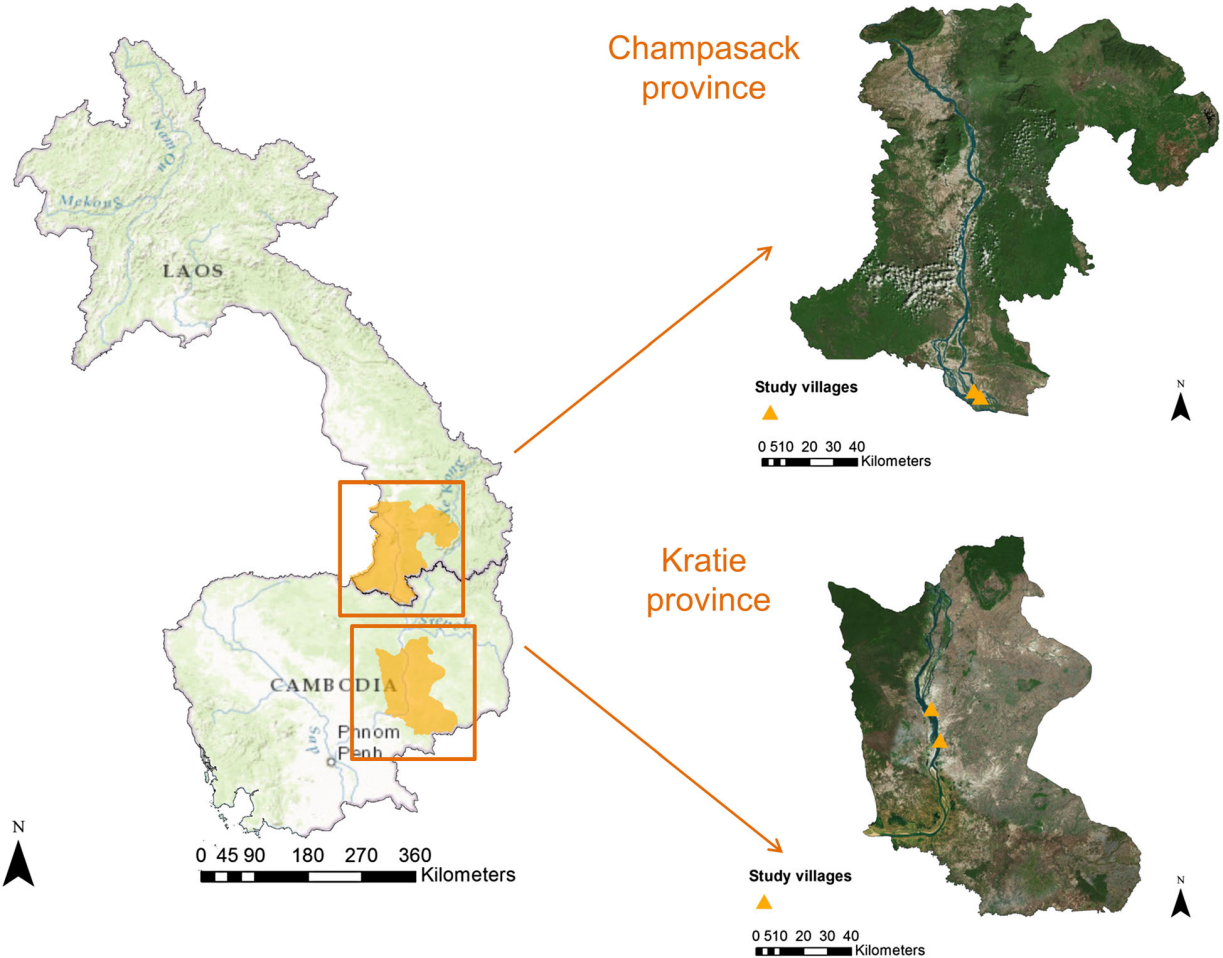
Lower course of the Mekong River at its crossing of the border between Lao PDR and Cambodia indicating the study area

Human schistosomiasis is caused by any of six species of blood flukes, namely *Schistosoma mansoni*, *S. japonicum*, *S. haematobium*, *S. mekongi*, *S. intercalatum* and *S. guineensis* [1]. The latter three species are not only in a clear minority but are also geographically restricted. *S. intercalatum* is endemic along part of Congo River and *S. guineensis* is found in lower Guinea on the African continent, while *S. mekongi* exists in limited areas near the border between Lao People’s Democratic Republic (Lao PDR) and Cambodia. Transmission of *S. mekongi* is highly focal [2, 3] with the overall distribution delineated by environmental variables suitable for the intermediate host snail *Neotricula aperta* [4]. The at-risk population is estimated at around 50,000 households comprising an estimated 150,000 people [2] (Fig. 1). Infection and re-infection in the endemic areas sustain the severe, chronic consequences of schistosome infection with its various complications [5]. Due to their high level of water contact, children are at the highest risk, which might result in retardation of growth and cognitive development.

The World Health Organization (WHO) roadmap for elimination of neglected tropical diseases (NTDs) [6] and the Regional Action Plan for NTDs in the Western Pacific Region for 2012–2016 issued by WHO’s Western Pacific Region Office (WPRO) [7] recommend targeting schistosomiasis mekongi for elimination. Delineation of infection occurrence based on valid documentation is a necessary step to reach this goal and success depends crucially on the availability of highly sensitive diagnostic techniques providing non-equivocal prevalence values in remaining endemic pockets. In mass deworming campaigns, schistosomiasis is treated with oral single-dose praziquantel (40 mg/kg body weight) since the early 1980s when this drug was introduced [8]. The current approach in communities affected by *S. mekongi* consists of preventive chemotherapy targeting at-risk populations (e.g. the entire population of villages along the Mekong) without prior diagnosis, complemented with the distribution of information, education and communication (IEC) packages and improvement of water, sanitation and hygiene (WASH) whenever resources allow [9]. The next stage now being considered is the elimination of this infection as a public health problem. Given its restricted distribution, eradication of *S. mekongi* might be envisaged. However, the preventive chemotherapy programmes implemented in endemic areas in Cambodia and Lao PDR make individuals harbouring mainly light-intensity infections likely to be missed by the standard and widely used Kato-Katz thick smear technique [10], resulting in imprecise assessment of the impact of preventive chemotherapy and other interventions. The solution lies in modifying the methodology applied according to the prevailing diagnostic need [11], which obliges assays to be more sensitive and specific when priorities shift from control of morbidity to interruption of transmission followed by surveillance [11, 12].

Apart from egg deposition, schistosome worms excrete (regurgitate) a number of different antigens into the host’s blood. The circulating anodic antigen (CAA) and its cathodic counterpart (CCA), described by Deelder and colleagues as early as in 1976 [13], are the most well-studied ones. Their detection in serum and urine has been followed up with continuously improving techniques, e.g. by de Jonge et al. [14], van Lieshout et al. [15], van Dam et al. [16] and Corstjens et al. [17]. Importantly, detection of the antigens in either blood or urine is evidence for an ongoing active infection as both antigens are subject to rapid renal clearance from the human circulation [13]. On the other hand, stool examination is marred by the problem that schistosome eggs can be detected up to several weeks after cure [14].

Diagnostic assays should preferably be applied in the field providing results at the point-of-care (POC) to allow appropriate test-and-treat approaches. Diagnostics utilizing the user-friendly, rapid-test platform based on lateral-flow (LF) immunochromatography are well suited for this type of test protocols [18]. As an alternative to egg detection in stool samples, a rapid POC assay for CCA detection in urine (POC-CCA) was developed for *S. mansoni* infection [16]. The POC-CCA assay is a visually read field assay, which takes about 20 min to perform and which does not require any equipment except the disposables provided with the kit. The colour intensity of the test line on the LF strip has a correlation to the number of eggs in the stool sample investigated [19, 20] and the read-out is at least as sensitive as duplicate Kato-Katz thick smears and considerably less laborious [21–23]. Although it was specifically developed for detection of *S. mansoni* infections, it has been shown to be suitable for other intestinal schistosomiasis-causing species such as *S. mekongi* and *S. japonicum* [24]. In order to increase sensitivity and wider applicability to other schistosome species, another LF-based test that detects CAA specifically and is based on the luminescent up-converting phosphor (UCP) reporter technology has been developed [17]. This test is referred to as UCP-LF CAA and includes different formats, depending on the matrix and sample volume used for testing [25]. The UCP-LF CAA test provides an assay applicable for all known *Schistosoma* species (including veterinarian ones) and is assumed to allow detection down to the level of a single worm pair while maintaining 100% specificity [25]. The CAA concentration is considered a good proxy for the number of worms present in the host [17]. This approach has been shown to work for *S. mansoni* [26] and *S. haematobium* infections [27, 28] as well as for infections by *S. japonicum* [29] and *S. mekongi* [24]. In the People’s Republic of China, the UCP-LF CAA assay demonstrated a *S. japonicum* prevalence of about 10 times higher than that estimated by triplicate Kato-Katz thick smears [29]. However, unlike the POC-CCA, the current UCP-LF CAA assay format is still a laboratory-based assay due to the need of centrifugation steps, hence not yet convenient for POC test-and-treat approaches.

Realizing that verification of transmission interruption requires a high level of sensitivity, we aimed to evaluate the new diagnostic techniques and to compare their results to the standard tools (e.g. Kato-Katz). We used the UCP-LF CAA assay formats to validate the POC-CCA test results. The POC-CCA test was expected to have higher sensitivity than the stool examination. As an extra control for sensitivity, *Schistosoma* serology based on an enzyme-linked immunosorbent assay (ELISA) was included as all active infections indicated by the POC-CCA assay should test positive with this approach unless the infection was only acquired very recently. It has to be noted that a positive test for specific antibodies is not only assured during active infections but indicate also former infections as antibody titres normally persist for a long time. This study compared a set of available assays to get a handle on the real prevalence and intensity of *S. mekongi* infections in the endemic enclaves in Cambodia and Lao PDR as the results should indicate a negotiable way forward with regard to elimination of the disease.

## Methods

### Study design, area and population

A cross-sectional study was conducted between February and April 2016 in *S. mekongi-*endemic villages in Lao PDR and Cambodia. Repeated stool examinations for intestinal helminth infections were conducted with particular emphasis on *S. mekongi* infection. Furthermore, urine and serum samples were obtained from each study participant to be tested for *Schistosoma* infection by the POC-CCA, UCP-LF CAA and ELISA assays.

Four villages, two in each of the endemic districts of Lao PDR and Cambodia, respectively, were selected. The villages Som VenOok and Ban Yai VeunSom in Khong district, Champasack province in southern Lao PDR were selected together with the villages Kbal Chuor and Sre Khoeurn in Kratié province in northern Cambodia (Fig. 1). The main occupation of the villagers was farming and fishing. All household members older than 6 years were enrolled. They were invited to fill in a questionnaire pertaining to demographic details and risk factors for infection, information on hygiene, disease knowledge and anthelminthic drugs taken during the last 6 months.

In Lao PDR, about 200 individuals living in Som VenOok and Ban Yai VeunSom, situated on islands in the Mekong River, were approached about the study. The study households were randomly selected from a list of households of the two villages. In Cambodia, according to the 2008 census, the total population was 2339 people (1602 in Kbal Chuor and 737 in Sre Khoeurn). Between 120 and 130 individuals were randomly selected from 30 to 35 households in each village.

### Sample collection and handling

#### Stool samples

Three stool samples were obtained from each participant during five consecutive days. Stool samples were subjected to examination by duplicate Kato-Katz thick smears (41.7 mg stool per smear) examined under a light microscope [30] by an experienced technician within 1 h after preparation on site in the study villages. Prior to microscopy, the thick smears were allowed to clear for 30 min after set-up. Eggs of all intestinal helminth species were counted and recorded for each species separately. The Kato-Katz thick smear examinations were performed directly in a convenient place in the study village (i.e. the village temple in Lao PDR; the village chief’s house in Cambodia).

#### Serum and urine samples

Blood samples were obtained from each participant, i.e. 5 ml venous blood (taken with vacutainers without anticoagulant) for serodiagnosis of *S. mekongi* infection and for the UCP-LF CAA assay. Urine samples (i.e. 10 ml urine) were obtained for CCA/CAA examination. Blood and urine samples were stored in cool-boxes at around 4 °C. In Cambodia, blood samples were centrifuged at Kratié Provincial Hospital a few hours after collection. Coagulated blood samples were centrifuged at 3000 rpm for 5 min and the upper part (serum) transferred to fresh tubes that were frozen and kept at −20 °C immediately after spinning, while the urine samples were directly frozen at −20 °C in the 15 ml-tubes they were collected in [29].

All samples were transferred frozen to a central national laboratory in Cambodia or Lao PDR and eventually shipped on dry ice to speciality laboratories at Swiss Tropical and Public Health Institute (Swiss TPH) in Basel, Switzerland and Leiden University Medical Center (LUMC) in Leiden, The Netherlands.

### Laboratory procedures

#### Detection of S. mekongi antibodies

*Schistosoma* serology was performed by ELISA at Swiss TPH using *S. mansoni* adult worm extract (AWE) and *S. mansoni* soluble egg antigen (SEA). Both *S. mansoni* antigens show cross-reactivity with antibodies elicited by other *Schistosoma* spp. (*S. haematobium*, *S. mekongi* or *S. japonicum*). The combination of both serological tests exhibits a sensitivity of 94.5% for *S. mekongi* infections and a specificity of 96% and 92% for AWE and SEA, respectively [31, 32].

AWE was prepared as described previously [31]. In brief, adult *S. mansoni* worms were homogenized in phosphate-buffered saline (PBS) of pH 7.2 containing 2-mM phenylmethylsulfonyl fluoride (PMSF). The extract was centrifuged at 80,000 g for 3 h at 4 °C and the pellet further extracted with PBS containing 1% Nonidet P40. After overnight incubation at 4 °C, the suspension was centrifuged again in the same way. After the supernatant had been concentrated and centrifuged at 15,300 g for 5 min at 4 °C, it was stored in aliquots at −80 °C until use. SEA was made from frozen *S. mansoni* eggs homogenized in PBS of pH 7.2 on ice and subsequently extracted for 3 h at 4 °C. The extract was centrifuged at 100,000 g for 2 h at 4 °C and the supernatant was stored in aliquots at −80 °C until use.

ELISA testing was carried out using Immulon 2HB plates (Thermo Labsystems; Beverly, MA, USA) coated with *S. mansoni* antigens in 0.05 M sodium carbonate buffer (pH 9.6) for 48 h at 4 °C. After washing with tap water containing 0.05% Tween 20, diluted sera (1:160 in PBS, pH 7.2, 0.05% Tween 20) were added to the plates that were incubated for 15 min at 37 °C. After additional washing steps, horseradish peroxidase conjugated goat-anti-human-IgG from Kirkegaard & Perry Laboratories (KPL) (http://kem-en-tec-nordic.com/kpl/) was added. Plates were incubated for 15 min at 37 °C, subsequently washed and o-phenylendiamine dihydrochloride (OPD) from Sigma (http://www.sigmaaldrich.com), diluted in 0.6-M sodium phosphate buffer of pH 5.0 supplemented with 0.03% H_2_O_2_, was added. The reaction was stopped with 8-M H_2_SO_4_ and the absorption read with a Thermo Scientific Multiscan FC reader (http://corporate.thermofisher.com) at 492 nm. The results of the ELISA tests were interpreted according to the cut-offs previously determined by receiver operating characteristic (ROC) analysis with sera from healthy Swiss blood donors, sera from *S. mansoni* infected patients and sera from patients with other parasitic infections, as described before [31, 32].

#### Detection of circulating schistosome antigens

This part of the study was carried out at LUMC. The POC-CCA test devices were obtained from Rapid Medical Diagnostics (Pretoria, South Africa) and tests were performed according to the manufacturer’s description. The amount of urine analysed per strip was 30 μl applied by pipette. Test results were visually interpreted, including distinction of trace-signals (weak colouration of the test line).

The UCP-LF CAA assay for urine was performed with 2 ml urine (the UCAA2000 assay format) as described earlier [25]. In short, 2 ml urine was extracted with 2-ml 4% (*w*/*v*) trichloroactetic acid (TCA). An Amicon centrifugal filtration device was used to concentrate the resulting clear supernatant (approximately 4 ml) to a final volume of 20–30 μl, of which 20 μl was analysed on UCP-LF CAA test strips using the wet-reagents format [25]. CAA concentrations were determined from standard series spiked in a negative urine sample and treated similarly to the clinical urine samples. The quality control (QC) cut-off threshold for singlet testing using the UCAA2000 wet-assay is 0.1 pg CAA per ml urine and the lower limit of detection = 0.05 pg/ml for testing performed in triplicate. Samples generating test results with a concentration between 0.05 and 0.1 pg per ml were counted as indecisive; samples with test results below 0.05 pg were considered CAA-negative [25].

The UCP-LF CAA assay for serum was performed with 0.5 ml serum (SCAA500) as described earlier [25]. The procedure was the same as described above with the difference that 0.5 ml TCA serum supernatant was concentrated to a final volume of 20 μl and the QC cut-off threshold was 1 pg CAA per ml serum with the lower limit of detection = 0.5 pg/ml. Samples generating test results with a concentration between 0.5 and 1 pg per ml were counted as indecisive; samples with test results below 0.5 pg were considered CAA-negative [25].

Note that the ultrasensitive assay format, specifically the SCAA500 test, is considered to allow identification of the majority of all active infections (including single-worm ones) [25]. In order to achieve the highest specificity, results were analysed considering the POC-CCA trace scores as well as the urine- and serum-CAA indecisive scores as negative. As this is a preliminary analysis, we decided to follow a conservative approach [25]. Generally, samples generating test results in the indecisive category would ideally require retesting with a larger sample volume to verify the true infection status.

### Statistical analysis

Demographic details of participants and their exposure to infection were obtained by questionnaire. Data were digitally collected using electronic tablets. The questionnaires and forms were developed in Commcare (http://www.commcarehq.org) format using the open data kit (ODK) programme (version 2.8) that was installed on the tablets for field data collection. Statistical analyses were performed in STATA version 13.1 (Stata Corp.; College Station, TX, USA). Only results from participants who had completed their questionnaires and stool examination were included in the final analysis.

The intensity of infection, expressed as eggs per gram of stool (EPG) obtained from Kato-Katz thick smear examinations were classified as light, moderate or heavy [33, 34]. The χ^2^-test was used to examine the association of categorical variables. The Spearman rank correlation test was used to correlate the results of the different diagnostic tests with each other. Spearman *r*- and *p*-values were reported. A *p*-value below 5% was considered statistically significant.

The *Schistosoma* ELISA assay (a marker of former or active infection) was composed of two separate assays, one based on AWE and the other based on SEA. For this study both these ELISAs were combined and an overall interpretation of both test results was applied. A result was interpreted as positive if at least one of the two ELISA tests was positive. A result was interpreted as inconclusive if both ELISA tests generated an inconclusive result. A result was considered negative if both ELISA were negative.

### Combined reference

We compared the diagnostic performance of all our stool, urine- and serum-based diagnostic tests to a combined reference. This consisted of a combination of test results of the Kato-Katz test, the urine-CAA test and the CAA-serum assay, all assays with a very high specificity, in particular as we followed a conservative approach accepting a relatively high cut-off threshold for the CAA tests. The sensitivity, specificity, positive and negative predictive values were calculated for all diagnostic tests based on this composite measure. For these calculations, traces and indecisive tests results of CAA and ELISA were taken as negative results. The urine- and serum-CAA tests were also combined into a total CAA outcome, which was deemed as positive when at least one of the two tests produced a positive outcome. This approach of comparing assays to a combined reference is a widely recognized method for assessment of diagnostic tests in the absence of a highly sensitive and specific ‘gold’ standard method and has been recommended by the WHO/TDR Diagnostics Evaluation Expert Panel [35].

## Results

### Study population

**Fig. 2:**
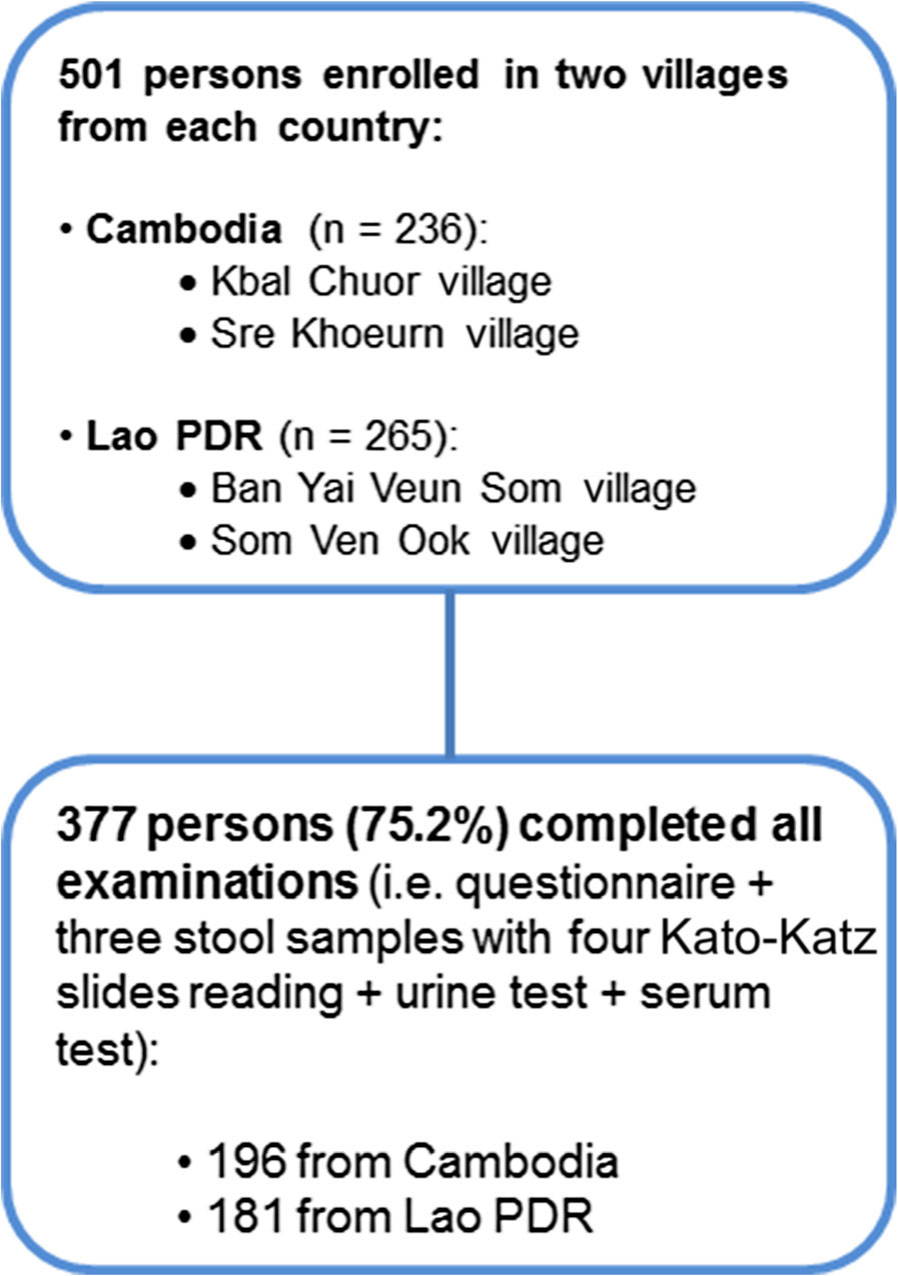
Study diagram

Data records could be completed for a total of 377 persons and they were included in the analysis carried out as shown schematically in Fig. 2. Of these, 196 (52.0%) were from Cambodia and 181 (48.0%) from Lao PDR. The age of the participants ranged from 6 to 79 years with a median of 25 years; slightly more females than males were enrolled (52.3% versus 47.8%). About half of the participants had finished primary school (53.3%); most of them were subsistence rice farmers and fishermen (61.3%). The social and demographic characteristics of study participants are summarised in Table 1.

**Table 1:** Demographic characteristics of the study participants

Parameter	Overall	Cambodia	Lao PDR	χ^2b^	*P-value* ^c^
	*n* (%)	*n* (%)	*n* (%)		
Number of subjects	377 (100)	196 (52.0)	181 (48.0)		
Age (years)					
Median (IQR)^a^	25 (12–44)	14 (11–35)	35 (15–47)	NA	NA
Sex					
Male	180 (47.8)	101 (51.5)	79 (43.7)		
Female	197 (52.3)	95 (48.5)	102 (56.4)	2.3	0.126
Age group (years)					
≤ 9	40 (10.6)	25 (12.8)	15 (8.3)		
10–16	116 (30.8)	82 (41.8)	34 (18.8)		
17–36	92 (24.4)	43 (21.9)	49 (27.1)		
37–50	68 (18.0)	22 (11.2)	46 (25.4)		
≥ 51	61 (16.2)	24 (12.2)	37 (20.4)	33.5	<0.001
Educational level					
Illiterate	11 (2.9)	0	11 (6.1)		
Primary school	201 (53.3)	101 (51.5)	100 (55.3)		
Secondary school	134 (35.5)	95 (48.5)	39 (21.6)		
High school	23 (6.1)	0	23 (12.7)		
Technical school or higher	8 (2.1)	0	8 (4.4)	64.9	<0.001
Occupation					
Farmer/fisherman	231 (61.3)	101 (51.5)	130 (71.8)		
Student	146 (38.7)	95 (48.5)	51 (28.2)	16.3	<0.001

(*IQR*)^a^ Inter quantile range; ^b^comparison between countries; *NA*, not applicable,

### Egg detection

The status of the participants according to helminth infection intensity categories is shown in Table 2. Overall, *S. mekongi* infection prevalence was 6.4% (24/377) with a much higher prevalence of 12.7% (23 positives) in Lao PDR, compared to 0.5% (one positive) in Cambodia. The overall results for prevalence of other helminth infections, such as *O. viverrini*, hookworm, *Trichuris trichiura*, *Ascaris lumbricoides* and *Taenia* spp. were 50.4%, 28.1%, 3.5%, 0.3% and 1.9%, respectively. Significantly higher prevalence rates were found for *O. viverrini* (90.1%), hookworm (50.8%) and *Taenia* (3.3%) in Lao PDR. Multiparasitism was observed in both countries with much higher frequency in Lao PDR than in Cambodia. Table 3 shows infection intensity categories recorded as EPGs. All infections were found to be light in Cambodia, while a large number of the *O. viverrini* infections were of moderate intensity in Lao PDR; some heavy infections (4 out of 181) were also identified there.

**Table 2:** Prevalence of *S. mekongi, O. viverrini* and other helminth infections among all study participants according to Kato-Katz examination

Helminth species	Overall (%)	Cambodia (%)	Lao PDR (%)	χ^2a^	*P- value* ^a^
Number of subjects	377 (100)	196 (52.0)	181 (48.0)		
Trematode					
*Schistosoma mekongi*	24 (6.4)	1 (0.5)	23 (12.7)	23.5	<0.001
*Opisthorchis viverrini*	190 (50.4)	27 (13.8)	163 (90.1)	219.0	<0.001
Nematode					
Hookworm	106 (28.1)	14 (7.1)	92 (50.8)	88.9	<0.001
*Ascaris lumbricoides*	1 (0.3)	0	1 (0.6)	1.1	0.297
*Trichuris trichiura*	13 (3.5)	7 (3.6)	6 (3.3)	0.02	0.892
Cestode					
*Taenia* spp.	7 (1.9)	1 (0.5)	6 (3.3)	4.1	0.044
Multiparasitism					
No infection	157 (41.6)	150 (76.5)	7 (3.9)		
Single infection	115 (30.5)	42 (21.4)	73 (40.3)		
Double infection	90 (23.9)	4 (2.0)	86 (47.5)		
Triple infection	14 (3.7)	0	14 (7.7)		
Quadruple infection	1 (0.3)	0	1 (0.6)	228.1	<0.001

^a^Comparison between countries

**Table 3:** Intensity of helminth infections among the infected study participants according to Kato-Katz examination

Species/Type of infection	Overall (%)	Cambodia (%)	Lao PDR (%)
Number of subjects	377	196	181
*Schistosoma mekongi*			
Light infection	24 (100)	1 (100)	23 (100)
*Opisthorchis viverrini*			
Light infection	116 (61.1)	27 (100)	89 (54.6)
Moderate infection	70 (36.8)	0	70 (42.9)
Heavy infection	4 (2.1)	0	4 (2.5)
Hookworm			
Light infection	104 (98.1)	14 (100)	90 (97.8)
Moderate infection	2 (1.9)	0	2 (2.2)
*Ascaris lumbricoides*			
Light infection	1 (100)	0	1 (100)
*Trichuris trichiura*			
Light infection	12 (100)	6 (100)	6 (100)

### Antigen detection

In total, 377 urine and serum samples were tested for *S. mekongi* infection (Table 4). In the urine samples, the CCA- and CAA-based test formats detected *S. mekongi* infections in 21.0% and 38.7% of all subjects, respectively. Compared to Cambodia, both urine tests diagnosed a higher *S. mekongi* prevalence in Lao PDR: 23.8% versus 18.4% with respect to CCA, and 42.5% versus 35.2% with respect to CAA. In serum, the latter test format detected a 32.4% overall prevalence with a similar difference between the two countries as found with the urine samples, 26.0% for Cambodia versus 39.2% for Lao PDR.

**Table 4:** Diagnosis of *S. mekongi* infection using serum and urine samples (*n* = 377)

Type of sample/method	Overall (%)	Cambodia (%)	Lao PDR (%)	χ^2^	*P-value*
Urine					
POC-CCA					
Negative	174 (46.2)	97 (49.5)	77 (42.5)		
Trace	124 (32.9)	63 (32.1)	61 (33.7)		
Positive	79 (21.0)	36 (18.4)	43 (23.8)	2.4	0.308
UCAA					
Negative	206 (54.6)	110 (56.1)	96 (53.0)		
Indecisive range	25 (6.6)	17 (8.7)	8 (4.4)		
Positive	146 (38.7)	69 (35.2)	77 (42.5)	4.0	0.133
Serum					
SCAA					
Negative	240 (63.7)	133 (67.9)	107 (59.1)		
Indecisive range	15 (4.0)	12 (6.1)	3 (1.7)		
Positive	122 (32.4)	51 (26.0)	71 (39.2)	10.9	0.004
ELISA combined^a^					
Negative	115 (30.5)	76 (38.8)	39 (21.6)		
Equivocal	132 (35.0)	68 (34.7)	64 (35.4)		
Positive	130 (34.5)	52 (26.5)	78 (43.1)	16.7	<0.001
Combined Reference^b^					
Negative	203 (53.8)	121 (61.7)	82 (45.3)		
Positive	174 (46.2)	75 (38.3)	99 (54.7)	10.2	0.001

*AWE*, adult worm antigen; *SEA*, soluble egg antigen; ^a^either AWE or SEA positive; ^b^at least one of the three tests (UCAA, SCAA, Kato-Katz) positive

### Detection of *S. mekongi* antibodies

The combined results of the two ELISA tests were positive in 34.5% of study participants, with a more than 16% higher rate in Lao PDR than in Cambodia (43.1% versus 26.5%). For all the diagnostic tests performed, the positivity rates were statistically significantly higher in Lao PDR compared to Cambodia (Table 4).

### Analysis of tests using the combined reference

We defined an active infection as an individual found positive for CAA (in urine or serum) or with a positive Kato-Katz thick smear. Table 5 shows the calculated sensitivity and specificity and the predictive values of the urine and serum tests in relation to this composite measure. The combined CAA tests had the highest calculated sensitivity (93.7%), followed by the urine- (83.9%) and serum-CAA (70.1%) test. The combined ELISA tests had a calculated sensitivity of 52.9% and a specificity of 81.3% against this combined reference. Triplicate Kato-Katz and single POC-CCA had a comparatively low sensitivity of 13.8% and 24.1%, respectively, and a negative predictive value of 57.5% and 55.7%, respectively (Table 5).

**Table 5:** Diagnostic characteristics of the various tests to diagnose *S. mekongi* infection using a combined reference^a^

Method	ELISA^b^	Kato-Katz	POC-CCA	CAA	CAA	CAA
Target	Antibodies	Parasite eggs	Circulating antigens			
Sample	Serum	Faeces	Urine	Urine	Serum	Serum + urine
	(%)	(%)	(%)	(%)	(%)	(%)
Sensitivity	52.9	13.8	24.1	83.9	70.1	93.7
Specificity	81.3	100	81.8	100	100	100
PPV*	70.8	100	53.2	100	100	100
NPV**	66.8	57.5	55.7	87.9	79.6	94.9

*Positive predictive value; **Negative predictive value; ^a^Infection-positive by either egg- or CAA-positivity (serum and urine combined, assuming 100% specificity of the CAA result). ^b^For the ELISA, either AWE and/or SEA positive was considered positive

### Analysis with respect to age and sex

Table 6 shows the positivity rate of the different diagnostic tests in relation to sex and age-groups. In general, all tests showed a higher positivity rate in males. A peak of positivity can be observed for the CAA tests in the age group 10–16 years. The ELISA results did not decrease with age to the same extent which could be explained by persistence of antibody titres for long time even after cured infections.

**Table 6:** Sex and age distribution of *S. mekongi* infection: results of various approaches

Method	ELISA	Kato-Katz	POC-CCA	CAA	CAA	CAA
Target	Antibodies	Parasite eggs	Circulating antigens			
Sample	Serum	Faeces	Urine	Urine	Serum	Serum + urine
	No. (%)	No. (%)	No. (%)	No. (%)	No. (%)	No. (%)
Sex						
Male	68 (37.8)	15 (8.3)	39 (21.7)	71 (39.4)	65 (36.1)	80 (44.4)
Female	62 (31.5)	9 (4.6)	40 (20.3)	75 (38.1)	57 (28.9)	83 (42.1)
Age group (years)						
≤ 9	3 (7.5)	1 (2.5)	6 (15.0)	8 (20.0)	6 (15.0)	9 (22.5)
10–16	44 (37.9)	2 (1.7)	25 (21.6)	56 (48.3)	53 (45.7)	64 (55.2)
17–36	36 (39.1)	10 (10.9)	23 (25.0)	39 (42.4)	27 (29.4)	42 (45.7)
37–50	25 (36.8)	6 (8.8)	9 (13.2)	22 (32.4)	19 (27.9)	24 (35.3)
≥ 51	22 (36.1)	5 (8.2)	16 (26.2)	21 (34.4)	17 (27.9)	24 (39.3)

### Correlation analysis

**Fig. 3:**
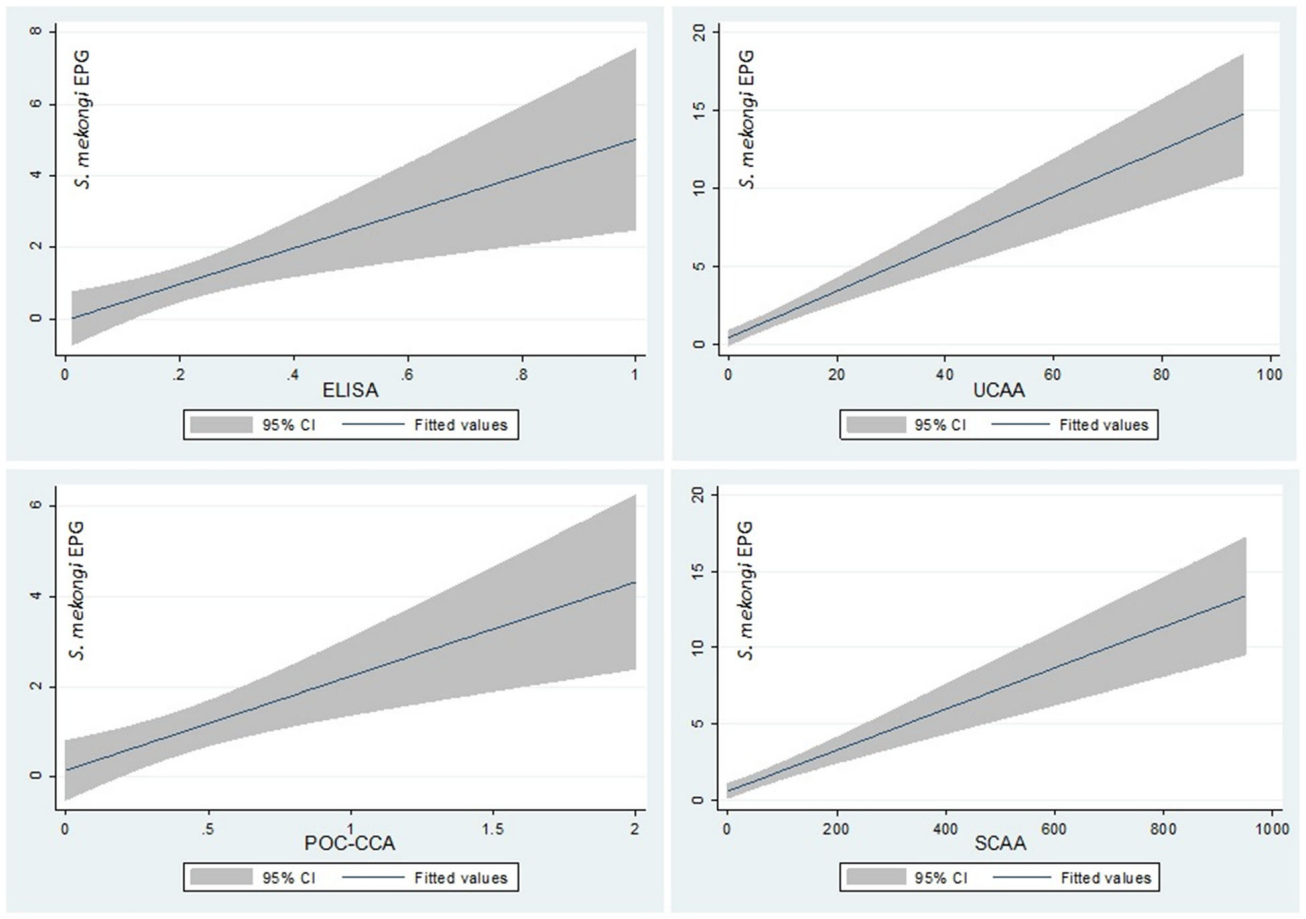
Correlation between combined ELISA (*top left*), POC-CCA (bottom left) and urine (*top right*) and serum CAA (*bottom right*) and infection intensity of *Schistosoma mekongi* (eggs per gram of stool)

Correlation analysis of the different diagnostic tests showed positive and statistically significant correlations between urine- and serum-CAA (*r* = 0.64, *p* < 0.001) and combined ELISA tests with serum-CAA (*r* = 0.55, *p* < 0.001) and urine-CAA (*r* = 0.38, *p* < 0.001). Furthermore, weakly positive but statistically significant correlations were detected between the infection intensity results of Kato-Katz and ELISA (*r* = 0.14, *p* = 0.005), POC-CCA (*r* = 0.12, *p* = 0.017), and urine (*r* = 0.11, 0.005) and serum-CAA (*r* = 0.17, *p* = 0.001) (Fig. 3). The correlation of the POC-CCA test results with the other tests were all weakly positive but statistically significant for urine-CAA (*r* = 0.15, *p* = 0.003) and serum-CAA (*r* = 0.14, *p* = 0.005). The correlation between the test results of POC-CCA and ELISA were weakly positive but not statistically significant (*r* = 0.09, *p* = 0.083).

## Discussion

The implementation of preventive chemotherapy has decreased schistosomiasis morbidity in endemic countries worldwide, including *S. mekongi* affected areas in Cambodia and Lao PDR [36, 37]. The current lower intensity of disease, however, is a compelling fact to recommend replacing stool examination using Kato-Katz with more sensitive diagnostic tools. Serology based on antibody detection is a helpful adjunct, but in order to determine cure and the level of worm burdens detection, assays based on the detection of circulating antigens are required. This approach has been successfully used for all major schistosome species showing that it is 10–20 times more sensitive than standard stool microscopy [17].

WHO recommends targeting schistosomiasis mekongi for elimination as the endemic areas are very limited and both stool examination according to Kato-Katz and antibody testing using ELISA serology indicate low intensity of disease after several rounds of preventive chemotherapy [6]. However, as has already been shown in the People’s Republic of China, highly sensitive tests for schistosome circulating antigens give considerably higher prevalence results than Kato-Katz [29]. With the proof-of-principle of testing for excreted antigens in the urine shown for *S. mekongi* [24], it was now felt that a field study in the endemic areas in southern Lao PDR and northern Cambodia would be warranted to establish this approach. In contrast to antibody detection, the Kato-Katz stool examinations along with the tests for circulating schistosome antigens (POC-CCA and UCP-LF-CAA) are all indicators of active infections. Antibody titres can persist for very long time after cure and therefore serology is not suitable for assessing treatment outcomes or as single diagnostic approach for detection of active infections.

In the field, detection of active infection and cure are all highly important, particularly when moving from control of a disease to transmission interruption and elimination. It is equally important for the individual patient. While the better sensitivity of antigen detection compared to Kato-Katz is obvious, it is also clear that CAA detection (both in serum and urine) performs much better than CCA. These results are in agreement with previous reports for *S. japonicum* and *S. mekongi* [24, 29, 38].

The advantage of the POC-CCA test is that it is a standardized urine test applicable in the field without the need for any extra equipment (fulfilling all ‘ASSURED’ characteristics). It has been mainly and widely validated for *S. mansoni* detection, but shows limited use for the other schistosome species [23]. However specificity is limited to some extent, because CCA has epitopes common with certain human components (Lewis-X structures) that sometimes end up in the urine causing false positive reactions [39]. The UCP-LF CAA test, on the other hand, is applicable for all schistosome species and for various human liquid samples, such as urine and serum, as well as potentially saliva [25]. In contrast to the POC-CCA assay, the UCP-LF CAA test format is not yet commercially available nor is its current format applicable for POC application because of a sample preparation procedure and the use of an UCP strip-reader. While the cost of the former is US$ 1–1.5 per test, that of the latter, being a manual laboratory test, is at least 10-fold higher. However, as shown here, the UCP-LF CAA test does display a superior sensitivity by concentration of the clinical sample and may therefore detect single-worm infections [25]. Still, as our results show that the POC-CCA assay is applicable for field diagnosis of *S. mekongi*, this assay should be the approach of choice for schistosomiasis diagnosis in Lao PDR and Cambodia with the current infrastructure.

We found a strong correlation of the test results of the urine and serum CAA tests and ELISA, while the correlations between the two CAA tests and the Kato-Katz and POC-CCA were weaker. These observations are consistent with previous studies in the People’s Republic of China [29] and elsewhere [28, 40] and are largely a reflection of the different sensitivities of these diagnostic tests.

It should be mentioned that the results presented here are interpreted rather conservatively with respect to the cut-off threshold, leaving the POC-CCA trace scores and the UCP-LF CAA indecisive values as negatives. A more detailed comparison of the different assays using e.g., latent class analysis may shed a better insight in the actual status of trace and indecisive samples. Such additional analyses, incorporating also a quantitative analysis of the POC-CCA results using a gold strip reader, are being planned.

In agreement with previous evaluations of the various assays for circulating schistosome antigens in areas endemic for other schistosome species, we found that the POC-CCA is both more rapid and more sensitive than multiple Kato-Katz thick smears. In the present study, the number of positives identified by POC-CCA was significantly higher than those found by Kato-Katz in both counties. These results are in accordance with published results which showed that POC-CCA prevalence was between 1.5- and up to 6-fold higher than Kato-Katz prevalence estimates in areas with low infection intensity [23]. The comparable cost levels per determination for POC-CCA and Kato-Katz [41, 42] should not prevent the application of the rapid test in national schistosomiasis control programmes. Furthermore, people are more likely to provide urine samples than any other type of sample, leading to higher compliance.

While eggs continue to be excreted by the host for a few weeks after cure, both CCA- and CAA-levels drop quickly, sometimes turning negative within 1 week after treatment [40, 43], making this approach a promising tool to monitor drug efficacy. The sensitivity of CCA-based tests is not as high as what the UCP-LF CAA assay or what DNA-based detection methods can offer [44, 45], while the ultrasensitive SCAA500 format of the UCP-LF CAA test surpasses PCR in sensitivity [46, 47]. As many different diagnostic assay systems are now available, planning to assess geographic areas potentially endemic for schistosomiasis, multiple diagnostic approaches should be compared taking into account modelling and statistical methods in combination with knowledge how biological systems operate [28, 48].

## Conclusion

Where low egg counts are most common, such as in areas characterised by low endemicity slated for elimination, the sensitivity and specificity of diagnostic tests must be taken into account when deciding which approach to choose. CCA-based assays are already available for use in the field, but tests targeting CAA still need the laboratory due to some of the sample preparation steps. Although the latter approach is the most sensitive antigen test, it would still be useful to apply POC-CCA testing for screening. While the results presented here will be subjected to further analysis, it would be useful to start planning for wider testing including application of geographical information systems to establish the real boundaries of the areas endemic for *S. mekongi*, prevalence and intensity of disease before moving on to transmission control and eventual elimination of the disease in Cambodia and Lao PDR.

## Additional file

Additional file 1: Multilingual abstracts in five of the six, official working languages of the United Nations. (PDF 902 kb)
