# Supplementary material for: Comparison of novel and standard diagnostic tools for the detection of Schistosoma mekongi infection in Lao People’s Democratic Republic and Cambodia
Source: Infect Dis Poverty. 2017 Aug 10;6:127. doi: 10.1186/s40249-017-0335-x (PMC5550959; doi:10.1186/s40249-017-0335-x)
Supplement: Additional file 1: — Multilingual abstracts in five of the six, official working languages of the United Nations. (PDF 902 kb) [file 40249_2017_335_MOESM1_ESM.pdf]

Translation of the abstract into the five official working languages of the United Nations

مقارنة بين طرائق التشخيص الجديدة والقياسية للكشف عن عدوى البلهارسيا الميكونغية في جمهورية لاو الديمقراطية الشعبية وكمبوديا

خلاصة البحث

الخلفية العلمية: نظرا للتوزيع المحدود للبلهارسيا الميكونغية في مقاطعة واحدة في جمهورية لاو الديمقراطية الشعبية ومقاطعتين في كمبوديا، معا مع التطور في برامج المكافحة الوطنية التي تهدف إلى الحد من الإصابة بالأمراض وانتشار العدوى، فإنه يمكن القضاء على البلهارسيا الميكونغية.مع ذلك، فهناك حاجة إلى طرائق تشخيصية حساسة لتحديد تحقق القضاءعليها. تم مقارنة العديد من طرائق التشخيصية القياسية والجديدة في المناطق الموبوءة بالبلهارسيا الميكونغية.

طرق البحث: تم تقييم انتشار وشدة العدوى بالبلهارسيا الميكونغية في 377 مشاركا في الدراسة من أربع قرى في المناطق الموبؤة في جمهورية لاو الديمقراطية الشعبية وكمبوديا بطريقة كاتو-كاتز لفحص البراز، الكشف عن الأجسام المضادة بتقنية مقايسة الممدص المناعي المرتبط بالانزيم (الأليزا) و كشف مستضد البلهارسيا الدوارة في الدم باختبار التدفق الجانبي. تم استخدام اختبارين حساسيين للكشف عن المستضدات الدوارة الموجبة والسالبة في المصل و الادرار.

النتائج: اوضح اختبار البراز المجهري ان نسبة الانتشار الكلية لطفيلي البلهارسيا الميكونغية بلغت 6.4% (حالة واحدة في كمبوديا و 23 حالة في جمهورية لاو الديمقراطية الشعبية)، بينما كانت نسبة انتشار ال *Opisthorchis viverrini* ، hookworm ، *Trichuris trichiura*، *Ascaris lumbricoides* و *Taenia* spp هي 50.4%، 28.1%، 3.5%، 0.3%، و 1.9% على التوالي. كشف اختباري المستضدات الدوارة الموجبة و المستضدات الدوارة السالبة ان نسبة الاصابة بالبلهارسيا الميكونغية هي 21.0% و38.7% على التوالي في نماذج ادرار المشاركين بالدراسة. وأظهراختبار المستضدات الدوارة الموجبة ان نسبة الانتشار 32.4% في نماذج المصل، في حين اظهر اختبار المستضدات الدوارة الموجبة لنماذج مصل الدم والأدرار نسبة انتشار 43.2%. لوحظ فرق بين موقعي الدراسة مع ارتفاع معدل الانتشار في عينات من جمهورية لاو الديمقراطية الشعبية.

الاستنتاجات: أظهرت نتائج اختبارات المستضدات الدوارة الموجبة والسالبة و مقايسة الممدص المناعي المرتبط بالانزيم (الأليزا) ارتفاع جوهري في تقديرات انتشارالبلهارسيا الميكونغية بالمقارنة مع نتائج اختبارالشرائح الكثيفة كاتو-كاتز لفحص البراز. لهذا قد تكون نسبة انتشار البلهارسيا الميكونغية في جمهورية لاو الديمقراطية الشعبية وكمبوديا قللت قيمتها إلى حد كبير أقل من السابق. لذلك لا تزال الحاجة الى جهود مستمرة للسيطرة وقطع انتقال البلهارسيا الميكونغية. ويجب التأكيد على الدور الحيوي للفحوصات التشخيصية ذات الحساسية العالية في المناطق الموبؤة لغرض التخلص منها.

老挝和柬埔寨湄公血吸虫感染检测的新诊断工具和标准诊断工具的比较

Youthanavanh Vonghachack, Somphou Sayasone, Virak Khieu, Robert Bergquist, Govert J. van Dam, Pytsje T Hoekstra, Paul L. A. M. Corstjens, Beatrice Nickel, Hanspeter Marti, Jürg Utzinger, Sinuon Muth and Peter Odermatt

摘要

引言：鉴于湄公血吸虫仅分布于老挝人民民主共和国（老挝）的一个省和柬埔寨两省，加上旨在减少发病率和患病率的国家防治项目的推进，湄公血吸虫病的消除似可实现。但是，需要敏感的诊断工具来确定是否已实现了消除。我们在湄公血吸虫流行区比较了几种标准诊断工具和新的诊断工具。

方法：在老挝和柬埔寨流行区的 4 个村庄，使用 Kato-Katz 法、基于酶联免疫吸附试验（ELISA）的抗体检测和基于侧流试验的血吸虫循环抗原检测调查了 377 名研究参与者，以评估湄公血吸虫的感染率和感染强度。使用两个高度敏感的检测系统来检测尿液和血清中的阴极和阳极循环抗原（CCA、CAA）。

结果：显微镜粪检结果显示，湄公血吸虫的总感染率为 6.4%（柬埔寨 1 例和老挝 23 例），而麝猫后睾吸虫（*Opisthorchis viverrini*）、钩虫、鞭虫、蛔虫和带绦虫的感染率分别为 50.4%、28.1%、3.5%、0.3%、1.9%。研究参与者的尿液样本检测发现湄公血吸虫的 CCA 和 CAA 阳性率分别为 21%和 38.7%。在血清样品中，CAA 检测结果显示感染率为 32.4%，而综合血清和尿液的 CAA 检测结果显示感染率为 43.2%。两个研究地点的不同点在于来自老挝的样本感染率更高。

结论：与 Kato-Katz 厚涂片法相比，根据 CCA、CAA 和 ELISA 的检测结果估算的湄公血吸虫感染率较高。以前的老挝和柬埔寨的急性湄公血吸虫病可能被大大低估了。因此，仍然需要持续的防控以打破疾病的传播。在计划消除的地区，高度敏感的诊断检测具有十分重要的关键性作用。

Translated from English version into Chinese by Yin-Long Li, edited by Pin Yang

CHINESE CENTER FOR DISEASE CONTROL AND PREVENTION
NATIONAL INSTITUTE OF PARASITIC DISEASES

Comparaison entre tests diagnostiques novateurs et standards pour la détection de l'infection à *Schistosoma mekongi* dans la République Démocratique Populaire du Laos et au Cambodge

Youthanavanh Vonghachack, Somphou Sayasone, Virak Khieu, Robert Bergquist, Govert J. van Dam, Pytsje T Hoekstra, Paul L. A. M. Corstjens, Beatrice Nickel, Hanspeter Marti, Jürg Utzinger, Sinuon Muth and Peter Odermatt

Résumé

Introduction: Compte tenu de la distribution géographique limitée de *Schistosoma mekongi* à une province de la République Démocratique Populaire du Laos (RDP Lao) et à deux provinces du Cambodge ainsi que les progrès réalisés par les programmes nationaux de lutte contre les helminthes afin de réduire infection et morbidité, une élimination de *S. mekongi* de la région semble atteignable. Cependant des tests diagnostiques sensibles sont nécessaires afin de d'affirmer cette élimination. Dans cet objectif, nous avons comparé plusieurs tests standards avec des outils diagnostiques novateurs dans la région endémique pour *S. mekongi*.

Méthodes: La prévalence et l'intensité de l'infection à *S. mekongi* ont été évaluées chez 377 participants d'une étude réalisée dans quatre villages dans les zones endémiques de RDP Lao et du Cambodge par un examen de Kato-Katz d'un côté, et par la détection des anticorps basée sur un essai immuno-absorbent (ELISA) de schistosomes et des antigènes circulants détectés par immuno-chromatographie sur bandelettes (Lateral Flow Test), de l'autre. De plus, deux tests ultra-sensibles pour la détection des antigènes circulants cathodiques (CCA) et anodiques (CAA) ont été utilisés dans l'urine et dans le sang des participants de l'étude.

Résultats: L'examen microscopique des prélèvements de selles détecte une prévalence d'infection à *S. mekongi* de 6.4% (un cas au Cambodge et 23 cas en RDP Lao). La prévalence de *Opisthorchis viverrini*, ankylostomes, *Trichuris trichiura*, *Ascaris lumbricoides* et *Taenia* spp. ont été de 50.4%, 28.1%, 3.5%, 0.3% et 1.9%. Dans les échantillons d'urine, les tests diagnostiques CCA and CAA ont identifié une infection avec *S. mekongi* de 21.0% et 38.7% des participants de l'étude. Sur le sérum, le test CAA a déterminé une prévalence de 32.4%, cependant la combinaison des tests CAA sur le sérum et dans l'urine a identifié une prévalence de 43.2%. La prévalence de l'infection a été significativement plus élevée en RDP Lao.

Conclusions: Les tests CCA, CAA and ELISA ont montré une prévalence d'infection par *S. mekongi* beaucoup plus élevée en comparaison avec le test Kato-Katz. L'infection active avec la schistosomiase en RDP Lao et au Cambodge a été considérablement sous-estimée préalablement. Par conséquent, un effort ferme est encore nécessaire pour interrompre la transmission de *S. mekongi*. Le rôle des tests diagnostiques ultra-sensibles est primordial dans les efforts d'éradication du parasite.

Сравнение новых и стандартного методов диагностики инвазии *Schistosoma mekongi* в Лаосской Народно-Демократической Республике и Камбодже

Youthanavanh Vonghachack, Somphou Sayasone, Virak Khieu, Robert Bergquist, Govert J. van Dam, Pytsje T Hoekstra, Paul L. A. M. Corstjens, Beatrice Nickel, Hanspeter Marti, Jürg Utzinger, Sinuon Muth and Peter Odermatt

Резюме

Введение: Принимая во внимание ограниченное распространение инвазии *Schistosoma mekongi* (*S. mekongi)* в одной провинции в Лаосской Народно-Демократической Республике (Лаос) и в двух провинциях Камбоджи, а также прогресс национальных программ по достижению контроля, направленных на снижение заболеваемости и распространенности инвазии, элиминация Меконгского шистосомоза представляется возможной. Однако, для верификации достижения элиминации требуется разработка чувствительных диагностических инструментов. Нами проведено сравнение нескольких стандартных и новых диагностических инструментов в эндемичных по инвазии *S. mekongi* районах.

Методы: Распространенность и интенсивность инвазии *S. mekongi* оценивали у 377 участников из четырех сельских населенных пунктов в эндемичных районах Лаоса и Камбоджи посредством исследования образцов стула методов Като-Кац, определения уровня антител методом иммуно-ферментного анализа (ИФА) и определения циркулирующих антигенов шистосом методом

латерального проточного иммуноанализа. Использовали две высокочувствительные тест-системы для обнаружения катодных (CCA) и анодных циркулирующих антигенов (CAA) в моче и сыворотке.
Результаты: Распространенность инвазии *S. mekongi* по результатам микроскопии стула составила 6,4% (один случай в Камбодже и 23 случая в Лаосе), в то время как распространенность инвазии *Opisthorchis viverrini,* анкилостомоза, *Trichuris trichiura, Ascaris lumbricoides* и *Taenia spp.* составила 50,4%, 28,1%, 3,5%, 0,3% и 1,9% соответственно. В образцах мочи CCA и CAA *S. mekongi* обнаружены у 21,0% и 38,7% участников исследования, соответственно. По результатам исследования CAA в образцах сыворотки распространенность *S. mekongi* составила 32,4%, в то время как при оценке CAA и в сыворотке и в моче распространенность достигла 43,2%. Установлено различие распространенности инвазии в двух регионах: наиболее высокие показатели регистрировались в Лаосе.
Выводы: По результатам использования CCA и CAA-тестов, а также ИФА установлены более высокие показатели распространенности инвазии *S. mekongi* в сравнении с результатами микроскопии стула по методу толстого мазка Като-Кац. Распространенность активного Меконгского шистосомоза в Лаосе и Камбодже ранее могла быть значительно недооценена. Следовательно, требуются постоянные мероприятия по достижению контроля в целях прекращения трансмиссии *S. mekongi*. Таким образом, использование высокочувствительных методов диагностики приобретает решающее значение в стратегии элиминации.

Comparativa de novedosas herramientas para la estandarización del diagnóstico de *Schistosoma mekongi* en la República Popular de Lao y en Camboya

Youthanavanh Vonghachack, Somphou Sayasone, Virak Khieu, Robert Bergquist, Govert J. van Dam, Pytsje T Hoekstra, Paul L. A. M. Corstjens, Beatrice Nickel, Hanspeter Marti, Jürg Utzinger, Sinuon Muth and Peter Odermatt

Resumen

Antecedentes: Dada la localizada distribución de *Schistosoma mekongi* en una sola provincia de Lao PDR y en solo dos provincias de Camboya, y junto con los avances de los programas nacionales de control destinados a reducir la morbilidad y la prevalencia de la infección, la eliminación de la esquistosomiasis parece factible. Aun así, siguen faltando herramientas de diagnóstico suficientemente sensibles para determinar si la eliminación definitiva ha sido conseguida. Nuestro objetivo en este trabajo, es comparar varias herramientas nuevas para el diagnóstico estándar de *S.mekongi* en áreas endémicas.
Métodos: La prevalencia e intensidad de la infección de *S.mekongi,* fue evaluada en 377 participantes residentes en cuatro aldeas endémicas en Lao PDR y Camboya. Las técnicas utilizadas fueron; Kato-Katz para el examen de heces, ELISA y detección de antígenos circulantes esquistosomáticos mediante pruebas de flujo lateral. También, se utilizaron dos sistemas de prueba altamente sensibles para la detección de antígenos circulantes catódicos (CCA) y anódicos (CAA) en orina y suero.
Resultados: La microscopía de heces reveló una prevalencia general de *S.mekongi* del 6,4% (un caso en Camboya y 23 casos en Lao PDR), mientras que en el caso de *Opisthorchis viverrini*, anquilostoma, *Trichuris trichiura*, *Ascaris lumbricoides* y *Taenia spp*, fue del 50.4%, 28.1%, 3.5%, 0.3% y 1.9%, respectivamente. En las muestras de orina, las pruebas de CCA y CAA se detectó infección con *S. mekongi* en el 21% y 38,7% de los participantes del estudio. En las muestras de suero, el ensayo CAA reveló una prevalencia de 32,4%, mientras que la combinación del ensayo CAA en suero y en orina reveló una prevalencia de 43,2%. Se observaron diferencias entre los dos lugares de estudio; con una mayor prevalencia en Lao PDR.
Conclusiones: Los resultados de CCA, CAA y ELISA mostraron estimaciones de prevalencia sustancialmente más altas que los frotis gruesos de Kato-Katz. La esquistosomiasis mekongi activa en Lao PDR y Camboya podría haberse subestimado considerablemente anteriormente. Estos resultados evidencian la necesidad de mejor medidas de control de la transmisión con *S.mekongi y la* importancia de implementar test de diagnósticos altamente sensibles para poder conseguir la eliminación.
